# Impact of body fat, body water content, and skeletal muscle mass index on peak salivary lactate levels after squat jump exercise in healthy non-athlete adult males

**DOI:** 10.1186/s13102-022-00482-6

**Published:** 2022-05-20

**Authors:** Satomi Okano, Hitomi Nishizawa, Joya Yui, Akinori Nakamura

**Affiliations:** 1 Department of Physical Therapy, Faculty of Health Sciences, Iryo Sosei University, 5-5-1 Chuodai Iino, Iwaki, Fukushima 970-8551 Japan; 2 School of Health Sciences, Faculty of Medicine, Shinshu University, 3-1-1 Asahi, Matsumoto, Nagano 390-8621 Japan; 3 Graduate School of Medicine, Shinshu University, 3-1-1 Asahi, Matsumoto, Nagano 390-8621 Japan; 4 Department of Neurology, Department of Clinical Research, National Hospital Organization Matsumoto Medical Center, 2-20-30 Muraimachi-minami, Matsumoto, Nagano 399-8701 Japan; 5 Third Department of Medicine, Shinshu University School of Medicine, 3-1-1 Asahi, Matsumoto, Nagano 390-0802 Japan

**Keywords:** Saliva, Lactate, Anthropometric characteristics, Squat jump, Non-invasive, Rehabilitation

## Abstract

**Background:**

In the rehabilitation and sports science fields, comprehensive assessment of the response to exercise is important for accurately prescribing exercise programs. Lactate is an important energy substrate that is frequently measured in clinical practice because it provides information on aerobic capacity. Salivary lactate, which can be measured non-invasively, has recently been focused on as an alternative to blood lactate. This study aimed to determine the combined effects of body fat, body water content, and skeletal muscle mass index on peak salivary lactate levels.

**Methods:**

Thirty-seven non-athletic males performed a squat jump exercise. Their salivary lactate levels were measured before, immediately after, and every 5 min after the exercise using a simplified device. We also assessed body composition. A linear multiple regression analysis was performed with peak salivary lactate levels as the dependent variable and body fat ratio, body water content, and the skeletal muscle mass index as independent variables.

**Results:**

The participants’ body fat ratio (positive effect; *p* = 0.001) and body water content (negative effect; *p* = 0.035) significantly affected peak salivary lactate levels. Skeletal muscle mass index tended to positively influence salivary lactate levels (*p* = 0.099), albeit not significantly. The adjusted R-squared value of the model was 0.312 (*p* = 0.001).

**Conclusions:**

The combined effect of body fat, body water content, and skeletal muscle mass index on peak salivary lactate levels was 31.2%. Better nutritional guidance may be effective in promoting weight loss and increasing body water content to improve aerobic capacity in the rehabilitation setting.

## Background

In the rehabilitation and sports science fields, comprehensive assessment of the response to exercise is important for accurately prescribing exercise programs and determining the effectiveness of training. Such responses to exercise included the metabolic [1], cardiovascular [2, 3], and respiratory [4] systems, and have been well documented in the literature. In hospitals, exercise prescriptions are commonly based on the anaerobic threshold [5] calculated from cardiopulmonary exercise testing (CPX), especially in the area of cardiac rehabilitation. Medical staff can obtain information on patients’ aerobic capacity using CPX. However, CPX systems are extremely large, expensive, and requires expertise, and so only a limited number of facilities are able to perform it. On the other hand, the point at which blood lactate (BLa) concentration increases exponentially during a graded incremental exercise test is defined as the lactate threshold (LT) and is often used in the area of sports science [6, 7]. Lactate is an important energy substrate [8, 9] that is produced by working skeletal muscle cells, circulates in the blood, and is metabolized in various tissues including the brain, heart, liver, and skeletal muscle [8, 10]. BLa is another measure of aerobic capacity [11]; as aerobic capacity improves, LT also increases. The higher LT that training provides means that energy metabolism during exercise is improved, which allows for higher endurance performance [12]. In contrast, for short-duration exercises such as squat jumps, sprints, and swimming as well as in sports in which measuring lactate during activity is difficult, it is common to evaluate aerobic capacity using peak lactate levels measured following an exercise task or trial. After the training period, if the peak lactate level is lower for the same load of exercise, aerobic capacity is considered to have improved. However, BLa measurement is slightly invasive, requiring blood sampling from the fingertip or earlobe [13, 14]. Salivary lactate (SLa) can be collected non-invasively, does not require special techniques for measurement [11], and has recently been considered an alternative method to BLa since both parameters reportedly trend in parallel during exercise. Segura et al. reported similar patterns of BLa and SLa transitions during the cycle ergometer maximal graded exercise test, which exhibited a positive correlation [15]. Santos et al. measured BLa and SLa at the 0 km point and at 6 km intervals of a 30 km run to reveal a positive correlation between BLa and SLa [11]. Moreover, Tekas et al. demonstrated a positive correlation between BLa and SLa before and after the maximum intensity Astrand treadmill test [16].

In addition to saliva, urine [17, 18] and sweat [19, 20] are also useful as specimens that can be collected non-invasively to determine lactate. However, it is difficult to obtain multiple urine samples in a short period of time, and it may not be possible to collect sufficient amounts of sweat to measure lactate levels reliably since perspiration varies greatly among individuals. Saliva-based measurements have higher clinical utility in these regards.

The greatest influence on lactate concentration is exercise intensity and working skeletal muscle cells, although other individual body characteristics may also affect lactate levels. For instance, adipocytes can produce lactate under anaerobic conditions, and the total amount of lactate produced by adipocytes is dependent on fat mass [10]. Regarding SLa, water transpiration due to exercise increases the amount of protein in the saliva [21] and may influence SLa levels. Whereas the above findings evaluate the relationship between a single physical parameter and lactate level, there have been no reports examining their combined effects in vivo. Since these multiple factors are considered to be acting simultaneously in the living body during rehabilitation, the purpose of this study was to determine the combined effects of body fat, body water content, and skeletal muscle on peak SLa levels.

## Methods

### Participants

A total of 37 healthy non-athletic males volunteered to participate in this observational study; their anthropometric characteristics are shown in Table 1. Informed consent was obtained from all participants after they were informed of the purpose and associated risks of this study.

The participants were instructed to refrain from strenuous exercise the day before the trial. On the day of the trial, food intake apart from water was prohibited 1 h before the experiment. All measurements were conducted in a well-ventilated room with a temperature of 25.0 ± 1.9 °C and humidity of 52.4 ± 5.7%. Based on national regulations and guidelines, all experimental protocols were reviewed and approved by the local Ethics Committee and conformed to the recommendations of the Declaration of Helsinki.

**Table 1:** The anthropometric characteristics of the participants

	Male (n = 37)
Age (years)	20 (19–21)^a^
Height (cm)	171.9 ± 5.6^b^
Body weight (kg)	60.7 (57.4–66.3)^a^
BMI (kg/m^2^)	20.7 (19.6–22.2)^a^
Skeletal muscle mass (kg)	30.5 ± 3.0^b^
SMI (kg/m^2^)	7.8 ± 0.6^b^
Body fat ratio (%)	12.1 (9.9–15.7)^a^
Body water content (kg)	39.7 ± 3.6^b^

*BMI1* Body mass index, *SMI* Skeletal muscle mass index

^a^Data are expressed as the median and interquartile range (IQR)

^b^Data are expressed as the mean ± standard deviation (SD)

### Squat jump (SJ) exercise

Prior to the trials, the examiner individually demonstrated and explained how to perform a standard SJ, which is often used to evaluate an athlete’s motor function for its simplicity and lack of specialized equipment [22, 23], and is also used during postoperative anterior cruciate ligament reconstruction rehabilitation [24]. Consistent with our previous studies [25, 26], a standard SJ included a squat, whereby bilateral lower limbs are spread to shoulder width, the knees flexed to 90°, with the trunk and lower legs parallel to each other, followed by a vertical jump as high as possible with the upper limbs swinging up and forward. The participants continued to repetitively perform SJs for 90 s at a constant tempo (46 bpm) guided by an electronic metronome (MA-1 SOLO METRONOME, KORG INC, Tokyo, Japan). In order to maximize their performance, the participants continued the task until the examiner signaled them to stop, without prior knowledge of the exercise duration. During the recovery phase after SJ, they rested in a sitting position.

### Measurement of salivary lactate level

Lactate Pro 2 (Arkray, Kyoto, Japan) is a commercially available device for BLa measurements. It is a point-of-care device with a measurable range of 0.5–25.0 mmol/L, where values < 0.5 mmol/L are displayed as “Lo,” and values > 25.0 mmol/L are displayed as “Hi.“ We previously investigated the use of the Lactate Pro 2 for measuring SLa. The intraclass correlation coefficient between the Lactate Pro 2 and a JCA-BM 8000 automatic analyzer was 0.773, indicating substantial convergent validity [26] and validating the use of this device to measure the SLa in the current study. SLa values were measured before exercise (Pre), immediately after exercise (Post_0_), and every 5-min during the recovery phase (Post_5_–Post_45_). Since we aimed to measure the peak value of SLa, measurements were stopped when the value of SLa decreased five consecutive times per participant. The subjects expectorated saliva onto a clean dish just before each measurement, and SLa levels were quantified by directly placing a Lactate Pro 2 sensor on the saliva samples. The participants were instructed to brush their teeth before SJ task and rinse their mouths once with water after the SJ to remove any oral debris [16, 27]. For the safety management of the participants, heart rate (HR; Polar M200, Polar Electro Japan, Tokyo, Japan) and the Borg CR10 scale were recorded in parallel to SLa [28].

### Body composition analysis

The body composition of each participant was measured using a high-precision body component analyzer (InBody 430, Inbody Japan, Tokyo, Japan) that can measure body components by bioelectrical impedance analysis, which measures the electrical resistance when small currents pass through the body [29]. The participants were instructed to stand barefoot on the device with light clothes on and grasp the handle with both hands in order to assess each body component. Body composition analyses were performed on the day of the trial, prior to the SJ exercise.

### Statistical analysis

The relationship between the levels of peak SLa and body fat ratio, body water content, and SMI was modeled using linear multiple regression analysis. For body fat, the ratio of body fat mass (kg) to body weight (kg) was used. As an indicator of skeletal muscle, SMI was used rather than skeletal muscle mass to eliminate the effect of the height of each participant and was calculated by dividing the total muscle mass of the extremities by the square of the height (m). Data for which a normal distribution was confirmed were expressed as the mean ± standard deviation (SD); those that were not were expressed as the median and interquartile range (IQR). All statistical analyses were performed using the PASW Statistics software (version 26.0, SPSS, Inc., Chicago, IL, USA), and the significance threshold was set at 0.05.

## Results

### Peak SLa levels after squat jump exercise

All subjects were able to complete 90 s of the SJ task at the speed of 46 bpm. Considering the data of the 37 participants, peak SLa levels were reached in 2 participants at Post_0_, 10 participants at Post_10_, 12 participants at Post_15_, 8 participants at Post_20_, 4 participants at Post_25_, and 1 participant at Post_30_. The peak SLa levels ranged from 0.7 to 7.7 mmol L, with an average value of 2.53 ± 1.49 mmol/L. The average values of the participant’s HR (123.1 ± 13.8 bpm) and Borg CR10 scale (6.8 ± 1.6) during the trial both reached maximum at Post_0_.

### Modeling SLa using body fat ratio, body water content, and SMI

The results of multiple regression analysis are presented in Table 2. The obtained multiple regression equation was as follows: SLa peak level = 0.167 + 0.157 × Body fat ratio + (− 0.227) × Body water content + 1.190 × SMI.

The analysis was performed using a forced entry method with SLa as the dependent variable and body fat, body water content, and SMI as the independent variables. The participant’s body fat ratio (positive effect; *p* = 0.001) and body water content (negative effect; *p* = 0.035) significantly affected SLa. SMI tended to positively affect SLa (*p* = 0.099), albeit not significantly. Body fat ratio was observed to have the greatest influence on SLa levels. The adjusted R-squared value of the model was 0.312 (*p* = 0.001). The variance inflation factor was 1.174–3.562, which reflects a considerably low multicollinearity between the variables. The residuals of this model were found to be normal.

**Table 2:** A model depicting the relationship between SLa peak values (mmol/L) and three anthropometric characteristics factors

Variable	Coefficient	95% CI	SE	Beta	t	*p* value
Intercept	0.167	− 5.913 to 6.247	2.988	–	0.056	0.956
Body fat ratio (%)	0.157	0.065 to 0.249	0.045	0.521	3.482	0.001**
Body water content (kg)	− 0.227	− 0.437 to − 0.018	0.103	− 0.556	− 2.205	0.035*
SMI (kg/m^2^)	1.190	− 0.238 to 2.618	0.702	0.442	1.695	0.099

*CI* Confidence interval, *SE* Standard error, *SLa* Salivary lactate, *SMI* Skeletal muscle mass index

R^2^ = 0.370, adjusted R^2^ = 0.312 (*p* = 0.001)

**p < 0.01; *p < 0.05

## Discussion

In this investigation, we examined the combined effects of body fat ratio, body water content, and SMI on peak SLa levels after a squat jump exercise towards the clinical adoption of SLa testing in the rehabilitation setting. The importance of this study is that SLa can be measured easily and non-invasively in the clinical setting as an indicator of aerobic capacity. Our results showed that peak SLa levels can be estimated using body parameters. Clinically, this will enable better guidance for nutritional management and body composition in patients during rehabilitation.

### Timing of peak SLa

BLa is known to increase immediately in response to exercise, whereas SLa increased 10–30 min after exercise in this study. Although the mechanism of this phenomenon could not be verified in the present investigation, several studies have reported similar results. Ohkuwa et al. measured BLa and SLa after 400-m and 3000-m run tests and showed that SLa peaked later than BLa [30]. Reer et al. measured BLa and SLa during a cycle ergometer test to reveal a clear delay in SLa as compared with BLa. One possible cause of this lag was considered the diffusion of lactate in the salivary glands [31].

### Salivary lactate was strongly influenced by body fat ratio and body water content

The variables in this study, body fat ratio and body water content, supported the results of previous studies, whereby Jansson et al. demonstrated lactate levels of obese men (894 ± 69 µM) to be significantly higher than those of lean males (738 ± 49 µM) [32]. Particularly, white adipocytes have been reported to produce large amounts of lactate [33, 34]. Furthermore, our study indicated that for every incremental increase in body fat ratio, a 0.157 mmol/L increase in SLa level can be expected (*p* = 0.001), which further strengthens a positive relationship between body fat and SLa. In contrast, we found a negative relationship between SLa and body water content, whereby every incremental increase in body water content was anticipated to cause a 0.227 mmol/L decline in SLa level (*p* = 0.035). Whilst there are limited studies on the effect of saliva flow or secretion rate on lactate levels, an increase in total salivary proteins due to the evaporation of saliva through exercise was reported by Lindsay et al. [21]. Thus, the increase in body water and water content of saliva are proportional, and thereby results in a decrease in the lactate concentration. Regarding SMI being adopted as an index for skeletal muscles, our results suggest that SLa levels are inclined to increase by 1.190 mmol/L for every incremental increase in SMI, which is consistent with a previous study that found skeletal muscle mass and SMI to be positively correlated with BLa reduction (*r* = 0.71) [25]. Moreover, Santos et al. demonstrated that there was a positive correlation (*r* = 0.772) between the absolute values of BLa and SLa measured every 6 km in a 30-km marathon race [11]. Based on these results, we hypothesized that SMI also affects SLa and extracted it as a dependent variable. While there was a positive relationship between peak SLa and SMI, there was no significant difference (*p* = 0.099) observed in this study. To date, there are no reports that show the absolute value of lactate (both in blood and saliva) to be directly related to skeletal muscle mass and/or SMI. Lactate during exercise is mainly produced in the skeletal muscle and is simultaneously taken up in the working muscle, heart, brain, and liver, and in skeletal muscle, is exchanged between white-glycolytic and red-oxidative fibers depending on the physiological state [9]. Further, lactate uptake is mediated by monocarboxylate transport (MCT) proteins, and the expression of MCT isoforms depends on muscle fiber type [35]. In our study, we measured total skeletal muscle mass and calculated SMI, but did not investigate the proportion of fast and slow muscle fibers or the expression level of MCTs in individuals, and therefore, it may be necessary to add molecular biological assessments related to lactate production, consumption, transport, and skeletal muscle mass for future studies. In addition, the design of this study only allowed the assessment of the relationship between peak SLa levels and physical parameters measurable in a clinical setting, without explicit consideration of the mechanism. The next step will be to examine how body fat and body water content, which were significantly associated with SLa as independent variables in this study, are circulated in the body and released into saliva after production by exercise. Based on the results of this study, however, we believe that training guidance can be provided to patients regarding body fat and body water content in clinical practice. Specifically, body fat ratio and body water content were shown to be positively and negatively related to SLa, respectively, indicating that weight loss and proper water intake could also be effective in improving aerobic capacity.

## Limitations

This study has several limitations. First, as we assessed only healthy young males it is unclear whether these results can be applied to females, as it has been reported that salivary hormone levels, ultrastructure, pH, flow rate, buffering capacity, and electrolyte levels change during the menstrual cycle [36]. In addition to sex differences, aging reduces saliva production and changes the surface tension and viscosity of saliva, which may also affect SLa levels [37]. Second, we performed measurements at 5-min intervals after observing that subjects had difficulty salivating after high-intensity SJ exercises in a pilot study. Further consideration measurement intervals are needed to ensure capture of peak SLa levels. It is also necessary to consider the absence of a consensus on stimulating saliva production, with various methods (acidic-based stimulation, chewing gum, gargling, etc.) used to date [21].

## Conclusions

This study demonstrated that body fat, body water content, and SMI impacted peak SLa by 31.2%. SLa increased significantly with higher body fat and decreased significantly with higher body water content. Clinically, these findings enable more tailored nutritional advice and suggest that patients who do not show sufficient SLa decreases despite participating in a rehabilitation program may require additional advice on dietary and water consumption habits. Further studies on the mechanism by which lactate produced by skeletal muscles is released into saliva during exercise and the relationship of BLa and circulating kinetics are warranted.

## Data Availability

Data are available upon reasonable request, as we are analyzing this data in another study. The data that support the findings of this study are available upon request from the corresponding author.

## Abbreviations

BLa: Blood lactate
LT: Lactate threshold
SLa: Salivary lactate
SMI: Skeletal muscle mass index
SJ: Squat jump
HR: Heart rate
MCT: Monocarboxylate transport
